# The Effect of Online Fitness Combining Dietary Intervention on Body Composition, Body Shame and Self-Esteem in Young Adults: A Randomized Controlled Trial

**DOI:** 10.3390/nu18091460

**Published:** 2026-05-02

**Authors:** Haoqin Chen, Pingqing Hu, Xiangang Yang, Yanchun Li

**Affiliations:** 1 China Institute of Sport and Health Science, Beijing Sport University, Beijing 100084, China; haoqinchen0@gmail.com; 2 School of Wushu, Beijing Sport University, Beijing 100084, China; 3 China Shooting College, Hebei Sport University, Shijiazhuang 050041, China; yangxiangang@hepec.edu.cn; 4 Beijing Key Laboratory of Sports Performance and Skill Assessment, Beijing 100084, China; 5 Key Laboratory for Performance Training & Recovery of General Administration of Sport, Beijing 100084, China

**Keywords:** time-restricted eating, mindful eating, web-based intervention, body composition, body shame, self-esteem

## Abstract

**Background**: Obesity is a major public health concern associated with adverse physical and psychological outcomes, including body shame (BS) and reduced self-esteem (SE). Lifestyle interventions incorporating dietary and exercise components, such as time-restricted eating (TRE), mindful eating, and structured physical activity, have shown promise; however, evidence on their combined effects within scalable, web-based formats remains limited. **Objective**: This study aimed to evaluate the effectiveness of a multi-component, web-based lifestyle intervention integrating TRE, mindful eating, and structured online exercise on body composition and psychological outcomes in young adults. **Methods**: In this pilot randomized controlled trial, 42 healthy young adults (age: 20.4 ± 1.6 years) were allocated to either an intervention group (*n* = 28) or a control group (*n* = 14). The intervention group followed an integrated program combining TRE, mindful eating principles, and guided online exercise sessions, while the control group received standard dietary and physical activity recommendations. Outcomes included body composition, anthropometric measures, BS (Weight- and Body-Related Shame and Guilt Scale), SE (Rosenberg Self-Esteem Scale), and eating behavior (Three-Factor Eating Questionnaire). **Results**: Significant group × time interactions were observed for body fat percentage (*p* < 0.001), fat mass (*p* = 0.001), and body mass (*p* = 0.025), with the intervention group demonstrating greater reductions compared with controls. BS scores significantly decreased in the intervention group, whereas no significant between-group differences were observed for SE (*p* > 0.05). Dietary adherence appeared higher than exercise adherence over the intervention period. **Conclusions**: A multi-component, web-based lifestyle intervention integrating TRE, mindful eating, and structured exercise may improve body composition and reduce BS in young adults. However, changes in SE were not observed over the short term. These findings support the feasibility of scalable digital lifestyle interventions, while highlighting the need for longer-term studies to clarify psychological outcomes.

## 1. Introduction

Obesity is a global public health challenge affecting all demographics and contributing to both physical and mental health issues, including body shame (BS) and low self-esteem (SE) [1,2]. In 2022, one in eight people worldwide was classified as obese [3], with rates in China showing 34.3% of adults as overweight and 16.4% as obese [4]. This growing burden is driven by economic growth and sociocultural factors [4], contributing to increased risks of noncommunicable diseases like cardiovascular disease and type 2 diabetes, as well as rising healthcare costs [5,6,7]. It is estimated that by 2035, the global economic impact of obesity will exceed $4.32 trillion annually. Additionally, the rise in body positivity awareness has highlighted the challenges of weight bias and stigma [8], which can further negatively impact SE and lead to heightened BS [9], especially among young adults [10]. Notably, individuals with a normal body mass index (BMI) but elevated body fat percentage (BF%)—a phenotype commonly referred to as normal-weight obesity (NWO)—may exhibit comparable metabolic risks, while the discrepancy between “normal” weight and higher adiposity may also contribute to body dissatisfaction and elevated BS.

Implementing dietary changes in China presents unique challenges due to the diversity of local cuisines and cooking methods, which complicates calorie calculation and adherence to strict dietary guidelines [11]. Time-restricted eating (TRE), a form of short-term fasting, emphasizes energy intake within a specific time window, avoiding the need for continuous calorie tracking [12]. This approach has shown potential in improving body composition by reducing the likelihood of overeating on non-dieting days [13,14]. Similarly, mindful eating, a practice that focuses on recognizing hunger and satiety cues, helps prevent unconscious overeating, particularly in overweight populations [15,16]. Both TRE and mindful eating avoid rigid calorie counting, making them culturally adaptable and feasible for Chinese populations. In addition, these approaches may enhance behavioral regulation and interoceptive awareness, potentially improving individuals’ perceived control over eating and reducing maladaptive eating patterns. Despite evidence supporting their independent benefits, their combined effects within a structured intervention remain underexplored [17,18].

High-intensity interval training (HIIT) has gained popularity for its time efficiency and effectiveness in weight management [19], as it can induce calorie burn even after exercise through excess post-exercise oxygen consumption (EPOC) [20]. HIIT’s minimal space and equipment requirements make it accessible, especially for young adults. When combined with fasting, HIIT has shown greater effectiveness in maintaining muscle mass and reducing fat compared to either method alone [21,22]. However, existing research in weight management has largely neglected mental health outcomes [23]. While weight loss has been associated with improvements in mental health factors such as depression and self-efficacy [23,24], studies on the impact of weight loss interventions on BS and SE are limited, particularly those that examine long-term effects [25,26].

Face-to-face lifestyle interventions have been shown to be effective in obesity prevention and treatment but are often impractical in middle-income countries like China due to high costs and resource inequalities [19]. In response, web-based interventions offer a promising alternative, providing scalable, accessible, and interactive support for weight management [27,28,29]. While many web-based programs focus on user engagement and health information [30], few implement a consistent, structured intervention across participants [28,31], and adherence remains a challenge, with most studies measuring adherence through login duration rather than actual activity participation [28,32].

This randomized controlled trial aimed to evaluate the effectiveness of a multi-component, web-based lifestyle intervention integrating mindful eating, TRE, and HIIT on body composition and psychological outcomes, including BS and SE, in young adults. Given the integrated nature of the intervention, the study was designed to assess its overall effects rather than to isolate the contribution of individual components. We hypothesized that the intervention group would achieve a reduction in BF%, stable or slightly increased muscle mass, and improvements in both BS and SE compared to the control group.

## 2. Materials and Methods

### 2.1. Participants

Forty-two healthy young adults (age: 20.4 ± 1.6 years; mass: 69.9 ± 14.3 kg; BF%: 35.5 ± 7.1%) from universities in Beijing, China, were recruited and randomly assigned to either an intervention group (*n* = 28) or a control group (*n* = 14) using computer-generated randomization in SPSS version 25.0.

An a priori power calculation was initially performed using G*Power (version 3.1) for a repeated-measures ANOVA (within–between interaction), assuming two groups, two time points, α = 0.05, and a moderate effect size. However, due to feasibility constraints in participant recruitment, the final sample size was determined pragmatically. A retrospective power estimation based on the achieved sample (*n* = 42) indicated that this study had approximately 70% power to detect moderate-to-large effects (f = 0.30), but may be underpowered for smaller or more subtle outcomes. Participants were eligible if they were aged 18–25 years, had a BMI between 18.5 and 34.9 kg/m^2^, and presented with elevated BF%, defined as ≥25% for men and ≥30% for women. Based on BMI and BF% criteria, participants were further classified into subgroups. NWO was defined as a BMI within the normal range (18.5–23.9 kg/m^2^) combined with elevated BF%, whereas participants with BMI ≥ 24.0 kg/m^2^ were classified as overweight/obese according to Chinese guidelines. Participants with orthopedic limitations, a history of stroke, eating disorders such as anorexia or bulimia, or medications affecting performance were excluded.

**Figure 1:**
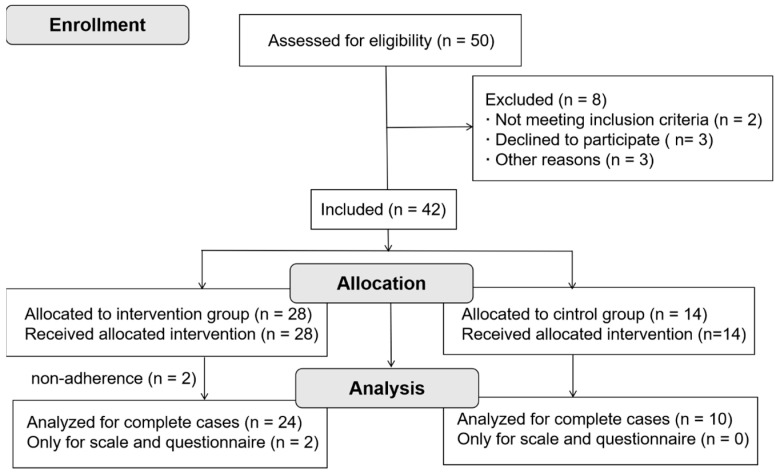
Progression of the participants through the trial.

This study was approved by the Sports Science Experiment Ethics Committee of Beijing Sport University and conducted in accordance with the Declaration of Helsinki. All participants provided written informed consent prior to participation. They were recruited on a voluntary basis and were not provided with any financial or material compensation for their involvement. Due to the nature of the intervention, participants were not blinded to group allocation. No follow-up assessments were conducted after the 8-week intervention period. Figure 1 illustrates the participant study flow.

### 2.2. Study Design and Intervention

The intervention lasted 8 weeks, with assessments conducted at baseline (one week prior to the intervention) and immediately after the intervention. This study was registered with the Chinese Clinical Trial Registry (ChiCTR2400094932). All participant interactions, including information dissemination, exercise and diet guidance, and feedback, were conducted online. The intervention group combined TRE with mindful eating and online fitness, while the control group followed the Chinese Dietary Guidelines (2022) and the Physical Activity Guidelines for Chinese (2021) without practical instructions.

#### 2.2.1. Exercise Protocols

Participants performed supervised online HIIT sessions three times per week on non-consecutive days over 8 weeks. Each session lasted approximately 20 min, including a 5 min warm-up and cool-down. The HIIT protocol consisted of bodyweight exercises (e.g., jumping jacks, squats, lunges, burpees) performed in timed intervals. Each exercise bout lasted 30 s, with work-to-rest ratios progressing from 2:3 in weeks 1–2 to 1:1 in weeks 3–8. The number of sets increased from 4 to 5 over the course of the intervention (Supplementary Table S1). Exercise intensity was guided using perceived exertion and video instruction, with participants encouraged to reach a moderate-to-vigorous effort level according to their individual fitness capacity. The program was structured into three progressive phases (familiarization, consolidation, and intensification), while allowing flexibility to accommodate individual differences in fitness and adherence.

The control group received standard recommendations for physical activity based on public health guidelines (i.e., 150–300 min/week of moderate-intensity or 75–150 min/week of vigorous-intensity aerobic activity, or an equivalent combination). No supervised exercise sessions or structured follow-up were provided, and participants were not monitored for adherence to these recommendations.

#### 2.2.2. Dietary Intervention

The dietary intervention incorporated an 8 h TRE window, during which participants were instructed to consume all daily caloric intake within a consistent 8 h period (e.g., 12:00–20:00). In addition, mindful eating principles were introduced, encouraging participants to initiate eating in response to physiological hunger cues and to maintain awareness of internal sensations of hunger and satiety during the eating period (Supplementary Table S2). During the 16 h fasting window, participants were instructed to consume only non-caloric beverages (e.g., water, black coffee, or unsweetened tea). The TRE schedule was implemented immediately without gradual progression. Participants received standardized educational sessions on TRE and mindful eating principles following baseline assessment, along with individualized weekly guidance throughout the intervention.

Dietary guidelines for the control group followed the principles of the Chinese Dietary Pagoda and eight specific recommendations.

### 2.3. Assessments

#### 2.3.1. Body Composition and Body Circumferences

Body composition was assessed using dual-energy X-ray absorptiometry (DXA) (GE Lunar Prodigy; GE Healthcare, Madison, WI, USA). The device was calibrated according to the manufacturer’s instructions prior to each testing session. Measurements were conducted under standardized conditions, with participants wearing light clothing (underwear only), without metal accessories, following an overnight fast (~12 h), and after voiding their bladder. All scans were performed by the same trained technician to minimize inter-operator variability. Body circumferences were measured using a tape measure (range 0–200 cm).

#### 2.3.2. Body Shame, Self-Esteem and TFEQ

Psychological outcomes were assessed using validated instruments. BS was measured using the Weight- and Body-Related Shame and Guilt Scale (WEB-SG) [33], which consists of 6 items rated on a 5-point Likert scale ranging from 1 (strongly disagree) to 5 (strongly agree). SE was assessed using the Rosenberg Self-Esteem Scale (RSES) [34], comprising 10 items scored on a 4-point Likert scale. Eating behavior was evaluated using the Three-Factor Eating Questionnaire (TFEQ-18) [35], which includes 18 items assessing cognitive restraint, emotional eating, and uncontrolled eating, each rated on a 4-point scale. All instruments demonstrated acceptable reliability in previous studies.

#### 2.3.3. Diet and Exercise

Adherence to dietary and exercise components was assessed using self-reported questionnaires. Dietary adherence was recorded daily at 21:00, including adherence to the 8 h eating window and subjective evaluation of eating behavior. Exercise adherence was recorded weekly, including frequency, type of activity, and perceived difficulty. Participants rated exercise completion on a 3-point scale (1 = difficult to complete; 3 = completed smoothly), which was used as an indicator of adherence. Weekly individualized e-counseling was provided to support compliance. Detailed questionnaires are provided in Supplementary Files S1 and S2.

### 2.4. Statistical Analyses

Statistical analyses were performed using IBM SPSS Statistics version 25.0 (IBM Corp., Armonk, NY, USA). Data are presented as mean ± standard deviation (SD). Baseline differences between groups were assessed using independent samples *t*-tests after confirming normality with the Shapiro–Wilk test. To evaluate the effects of the intervention, a two-way repeated-measures ANOVA (group × time) was conducted for primary outcomes, including BF%, BS, and SE. Effect sizes were reported using partial eta squared (η^2^), interpreted as small (0.01), moderate (0.06), and large (0.14). The internal consistency of the psychological scales (BS, SE, and TFEQ) was assessed using Cronbach’s alpha. Correlation analyses were performed using Pearson’s or Spearman’s coefficients, as appropriate, and were considered exploratory. Analyses were conducted on a complete-case basis, and missing data were not imputed due to the limited sample size. Statistical significance was set at *p* < 0.05.

## 3. Results

### 3.1. Changes in Body Composition and Circumferences

Table 1 details the baseline characteristics of participants. No differences were noted among the intervention groups for these variables.

**Figure 2:**
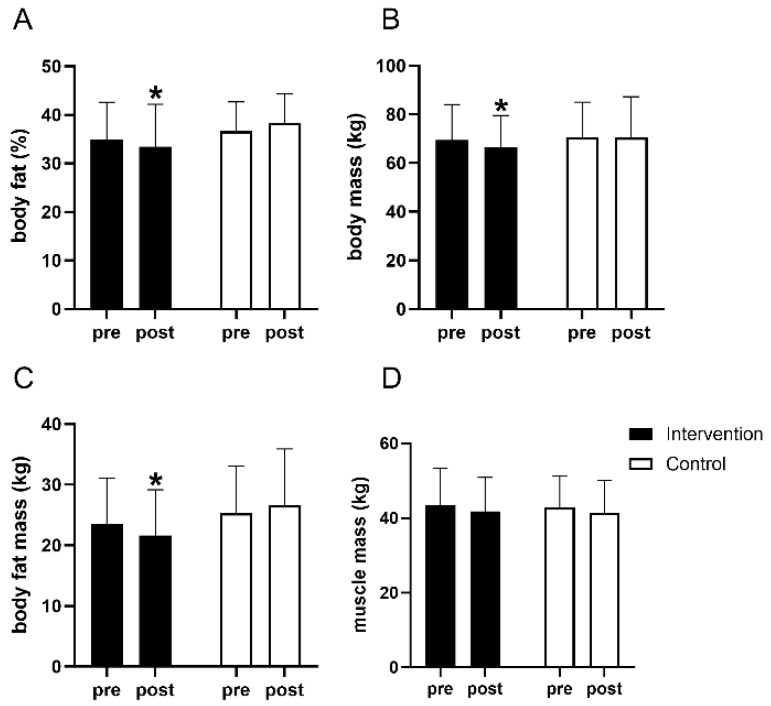
Body composition analysis. Changes in the intervention and control groups before (pre) and after (post) the 8-week intervention are presented for (**A**) body fat (%) (i.e., body fat percentage, BF%), (**B**) body mass, (**C**) body fat mass, and (**D**) muscle mass. Data are mean ± SD. * Significantly different from pre-values (*p* < 0.05).

After 8 weeks, a significant main effect of time (*p* < 0.001, partial η^2^ = 0.504) and a time–group interaction (*p* < 0.001, partial η^2^ = 0.350) were observed for BF% (Table 2). The intervention group showed a substantial reduction in BF% of approximately 6% (from 35.0 ± 7.7% to 33.43 ± 8.74%), while the control group remained stable (from 36.6 ± 6.0% to 38.33 ± 5.98%). This pattern extended to total body fat mass and body mass. Specifically, fat mass in the intervention group decreased by about 9% (from 23.59 ± 7.45 kg to 21.66 ± 7.52 kg), whereas the control group experienced a slight increase of 0.5% (from 25.32 ± 7.96 kg to 26.62 ± 9.29 kg). Furthermore, body mass showed a significant interaction effect (*p* = 0.025) and main effect over time (*p* = 0.001), with the intervention group seeing a ~3% decrease (from 69.5 ± 14.5 kg to 66.63 ± 12.91 kg) compared to a negligible decrease of 0.2% in the control group (from 70.7 ± 14.3 kg to 70.50 ± 16.82 kg). Muscle mass was maintained across both groups, with the minor changes from 43.44 ± 9.97 kg to 41.81 ± 9.12 kg in the intervention group, and from 42.98 ± 8.29 kg to 41.49 ± 8.66 kg in the control group (Figure 2).

Despite the absence of a statistically significant time–group interaction for the waist–hip ratio in the intervention group, significant gender–time interactions were evident, with notable effects on waist–hip ratio (interaction: *p* = 0.016; main gender effect: *p* = 0.001). Additionally, total body fat mass displayed a significant interaction (*p* = 0.033), but lacked a main gender effect. In contrast, BF% and body mass each showed a significant main gender effect (*p* = 0.002 and *p* = 0.004, respectively), without interactions. No BMI–gender interaction was observed in the intervention group; however, a significant interaction between BMI categories and time impacted the waist–hip ratio (*p* = 0.013). For participants in the intervention group, categorized as normal weight (*n* = 13), overweight and obese (*n* = 10), significant main effects were noted in total body fat mass, body mass, BMI, and waist–hip ratio (*p* < 0.001 for each, except waist–hip ratio at *p* = 0.014) (Table 3).

### 3.2. Body Shame and Self-Esteem

The internal consistency of the psychological scales was acceptable to good in the present sample, with Cronbach’s alpha coefficients of 0.885, 0.762, and 0.805 for BS, SE, and TFEQ at baseline, and 0.873, 0.842, and 0.833 at post-intervention, respectively. Significant time–group interactions were observed for both BS (*p* < 0.001) and SE (*p* = 0.002). BS exhibited a pronounced time effect (*p* < 0.001), unlike SE. Across all participants, BS and SE were moderately and negatively correlated at baseline (r = −0.54) and post-intervention (r = −0.44), suggesting that increasing BS was associated with decreasing SE. At baseline, this negative correlation was consistent across BMI-defined normal-weight individuals with elevated body fat (i.e., NWO) and those classified as overweight/obese based on BMI criteria (r = −0.55 and r = −0.54, respectively). By the study’s end, the correlation ceased for normal-weight individuals but strengthened for the overweight and obese (r = −0.77). Conversely, while males showed no significant correlations, females displayed stable moderate negative correlations throughout (r = −0.57 pre, r = −0.54 post), detailed in Table 3.

In the total sample (*n* = 34), no statistically significant correlations were observed between changes in any body composition variable and BS or SE, except for a moderate negative correlation between change in cognitive restraint of eating and SE (Spearman’s ρ = −0.42, *p* < 0.05). No other TFEQ subscale (emotional eating, uncontrolled eating) correlated significantly with either BS or SE in the total sample. Within the intervention group (*n* = 24), moderate positive correlations emerged between reductions in body circumference measures and BS: waist circumference% (r = 0.51, *p* < 0.05) and waist–hip ratio% (r = 0.44, *p* < 0.05). No significant correlations with SE were observed in this subgroup. All remaining correlations between body composition variables (including BF%, body fat mass, body mass, BMI) and BS/SE were non-significant across both the total sample and the intervention subgroup (*p* > 0.05).

### 3.3. Dietary and Exercise

Complete adherence data were available for 26 participants in the intervention group. Dietary adherence was assessed as the proportion of days participants reported following the prescribed 8-hour eating window. Exercise adherence was defined as the proportion of planned weekly sessions completed, based on self-reported logs. A moderate positive correlation was observed between overall dietary self-evaluation scores and adherence to the 8-hour eating window (r = 0.52, *p* = 0.007). No significant association was found between self-evaluation scores and reported hunger-related indicators. Additionally, self-evaluation scores were moderately negatively correlated with changes in BF% (r = −0.41, *p* = 0.050).

Regarding adherence levels, 85% of participants adhered to the 8-hour eating window for more than 70% of the intervention days, while 46% adhered for more than 80% of days. Additionally, 77% of participants reported experiencing physiological hunger cues for more than 80% of the intervention period. Exercise adherence showed greater variability. Over the 8-week intervention, 53% of participants exercised twice per week, and 35% exercised three times per week. Weekly exercise completion rates were as follows: 34% of participants achieved ≥70% completion, and 62% achieved ≥80% completion. Adherence to the prescribed HIIT sessions declined gradually over time: weekly adherence rates were 44%, 51%, 69%, 46%, 51%, and 57% during weeks 3-8. In the final three weeks, 41%, 33%, and 33% of participants engaged in alternative non-HIIT physical activities when unable to complete the structured sessions.

Given that adherence was assessed through self-reported measures, these findings should be interpreted with caution due to the potential for reporting bias.

## 4. Discussion

Our study aimed to explore how combining mindful eating principles, TRE and online fitness affects body composition, BS and SE in young adults. Over the 8-week period, reductions in fat mass, body mass, BF%, and BS were observed in the intervention group, with differences between groups over time. In addition, exploratory analyses identified associations between WC/WHR, gender, and BS. The study was not designed to isolate the effects of individual components or to establish causal relationships.

A recent meta-analysis reported fat mass and weight losses from combining exercise and intermittent fasting ranging from 0.21 to 1.23 kg and 2.61 to 3.44 kg, respectively [36]. In contrast, participants in our intervention group experienced fat mass reductions from 1.32 to 2.73 kg and weight losses from 1.15 to 3.16 kg. Furthermore, BF% in the intervention group significantly decreased compared to the control group, while muscle mass remained stable in both groups, in line with previous face-to-face studies [37]. The use of DXA to measure these anthropometric variables is noteworthy, as few web-based interventions employ such precise body composition assessments. Except for studies by Lisón et al., which used a body-fat analyzer [28], and Kohl et al., which used bioelectrical impedance scales [31], most web-based interventions rely on self-reported data [27,38,39,40]. This limits measurement accuracy and does not provide direct information on body composition changes.

In the present study, no significant between-group differences in WHR were observed. Exploratory subgroup analyses suggested potential interactions with gender and BMI categories within the intervention group. Similar variability in waist-related outcomes has been reported in previous studies [40], particularly in samples with mixed BMI classifications [41,42]. Given the small subgroup sizes and exploratory nature of these analyses, these findings should be interpreted with caution.

Significant group × time interactions were observed for both BS and SE; however, a clear reduction over time was observed only for BS. The absence of a significant time effect for SE is consistent with previous studies reporting variable responsiveness of SE to short-term interventions [43,44,45]. Correlation analyses indicated a moderate negative association between BS and SE at baseline and post-intervention, particularly among female participants [46]. These findings are in line with prior research suggesting a relationship between body-related perceptions and self-evaluative constructs. In addition, exploratory analyses showed associations between changes in WC and WHR and BS scores.

Adherence to the 8 h eating window was relatively high in this study, based on self-reported data. Associations were observed between adherence to the eating window and changes in BF%. However, adherence measures relied on self-report and were not objectively verified. No clear associations were observed between mindful eating-related indicators and changes in body composition. Given the multi-component design and lack of direct measurement of underlying mechanisms, the specific contribution of dietary or behavioral components cannot be determined. A difference in cognitive restraint eating between groups was observed, whereas previous studies have reported inconsistent findings during fasting interventions [47]. Notably, correlations were observed between changes in cognitive restraint and BS, indicating an association between eating behavior and psychological measures in this sample.

Regarding exercise, one-third of participants strictly complied with the intended frequency, and half followed the recommended sequence. Given that online fitness allows participants to select exercises of appropriate intensity, this level of compliance is consistent with previous reports in similar web-based interventions [28]. In the second phase, the number of participants following the prescribed plan increased during weeks 3–5 but declined in week 6, coinciding with the emergence of alternative self-selected exercises. Adherence to the prescribed HIIT sessions declined over time, with some participants switching to alternative forms of exercise in later stages of the intervention. As exercise type and intensity were not strictly controlled or objectively monitored, the specific contribution of HIIT to the observed outcomes cannot be determined.

This study has several limitations. First, the sample size was relatively small with an unbalanced 2:1 allocation. Although an a priori power calculation was conducted, recruitment constraints resulted in a smaller final sample. Retrospective estimation suggests adequate power to detect relatively large effects, but limited sensitivity for smaller or more subtle outcomes, particularly for psychological variables. The unequal allocation may further reduce statistical efficiency and precision of between-group comparisons. Second, the short intervention period (8 weeks) and absence of long-term follow-up preclude assessment of whether improvements in body composition or eating behaviors are sustained. Third, the sample’s narrow age range and unbalanced gender ratio limit generalizability. Fourth, reliance on self-reported adherence (dietary and exercise) introduces recall and social desirability bias; objective monitoring (e.g., accelerometry) was not used. Fifth, analyses were conducted on a complete-case basis; missing outcome data were not imputed, and we did not perform an intention-to-treat analysis. This may have introduced attrition bias and overestimated intervention effects. Sixth, the intervention is multimodal (TRE, mindful eating principles, online HIIT), making it difficult to isolate the contribution of each component. Moreover, adherence to prescribed HIIT varied considerably, with many participants switching to alternative exercises in later weeks—a form of intervention heterogeneity that limits causal conclusions about HIIT specifically. Seventh, the Hawthorne effect (participants’ awareness of being monitored) cannot be ruled out and may have enhanced adherence or reporting. Finally, declining compliance over time highlights the challenge of sustaining engagement in unsupervised web-based interventions.

Overall, the findings of this study should be interpreted as preliminary and descriptive. While changes in body composition and BS were observed alongside the intervention, the study design does not allow attribution of these changes to specific components or mechanisms. The use of DXA represents a methodological strength in outcome assessment. Future studies with larger samples, longer follow-up, and more controlled designs are required to confirm these observations and clarify potential mechanisms.

## 5. Conclusions

This pilot study examined associations between a web-based, multi-component lifestyle intervention and changes in body composition and psychological outcomes in young adults. Reductions in fat-related measures and BS were observed in the intervention group; however, given the exploratory design, small sample size, and lack of component isolation, these findings should be interpreted as preliminary and descriptive. No causal inferences can be drawn. Further research with larger samples, longer follow-up, and more controlled designs is required to verify these observations and to examine underlying mechanisms.

## Figures and Tables

**Table 1:** Baseline characteristics.

Variables	Intervention Group (*n* = 28)	Control Group (*n* = 14)	t (p)
Male (%)	32.1	21.4	
Age (yrs)	20.5 ± 1.8	20.3 ± 1.2	0.402 (0.690)
Body mass (kg)	69.5 ± 14.5	70.7 ± 14.3	−0.264 (0.793)
BMI (kg/m^2^)	24.4 ± 3.6	25.2 ± 3.5	−0.698 (0.489)
BF%	35.0 ± 7.7	36.6 ± 6.0	−0.720 (0.476)
Waist–hip ratio	0.80 ± 0.05	0.84 ± 0.07	−1.773 (0.093)
BS	18.00 ± 5.70	17.71 ± 6.98	0.142 (0.888)
SE	31.18 ± 4.11	30.43 ± 6.09	0.416 (0.682)

Data are presented as mean ± SD. ‘t (p)’ reports results from independent sample *t*-tests, applying Welch’s test when variances are unequal. § chi-square statistics.

**Table 2:** Two-way ANOVA results for body mass, fat mass, BF%, and BMI.

Predictor	Body Mass (kg)		Fat Mass (kg)		BF%		BMI (kg/m^2^)	
	η^2^	*p*	η^2^	*p*	η^2^	*p*	η^2^	*p*
Intercept	0.708	<0.001	0.387	<0.001	0.640	<0.001	0.820	<0.001
Time	0.281	0.001	0.467	<0.001	0.504	<0.001	0.270	0.002
Time × group	0.147	0.025	0.303	0.001	0.350	<0.001	0.139	0.030

η^2^ = partial eta squared.

**Table 3:** Two-way ANOVA results for time × gender and time × BMI category interactions in the intervention group.

Predictor	Body Mass (kg)		Fat Mass (kg)		Waist–Hip Ratio		BMI (kg/m^2^)	
	η^2^	*p*	η^2^	*p*	η^2^	*p*	η^2^	*p*
Time × gender	0.034	0.332	0.156	0.033	0.196	0.016	0.012	0.566
Time × BMI category	0.025	0.494	0.026	0.480	0.162	0.013	0.022	0.536

η^2^ = partial eta squared. BMI category: normal-weight (*n* = 13) vs. overweight/obese (*n* = 11) based on baseline BMI criteria. Gender: male vs. female.

## Data Availability

The data that support the findings of this study are not publicly available due to privacy and ethical restrictions but are available from the corresponding author upon reasonable request.

## Supplementary Materials

The following supporting information can be downloaded at: https://www.mdpi.com/article/10.3390/nu18091460/s1, Table S1: HIIT protocol details; Table S2: Core principles of the 16/8 TRE and mindful eating approach; File S1: Daily dietary adherence and satisfaction questionnaire; File S2: Weekly exercise adherence and satisfaction questionnaire.

## References

[1] Ahmed S.K., Mohammed R.A. (2025). Obesity: Prevalence, causes, consequences, management, preventive strategies and future research directions. Metabol. Open.

[2] Islam A., Sultana H., Nazmul Hassan Refat M., Farhana Z., Abdulbasah Kamil A., Meshbahur Rahman M. (2024). The global burden of overweight-obesity and its association with economic status, benefiting from STEPs survey of WHO member states: A meta-analysis. Prev. Med. Rep..

[3] Zhou B.F. (2002). Predictive values of body mass index and waist circumference for risk factors of certain related diseases in Chinese adults—Study on optimal cut-off points of body mass index and waist circumference in Chinese adults. Biomed. Environ. Sci..

[4] Pan X.F., Wang L., Pan A. (2021). Epidemiology and determinants of obesity in China. Lancet Diabetes Endocrinol..

[5] Okunogbe A., Nugent R., Spencer G., Ralston J., Wilding J. (2021). Economic impacts of overweight and obesity: Current and future estimates for eight countries. BMJ Glob. Health.

[6] Afshin A., Forouzanfar M.H., Reitsma M.B., Sur P., Estep K., Lee A., Marczak L., Mokdad A.H., Moradi-Lakeh M., Naghavi M. (2017). Health Effects of Overweight and Obesity in 195 Countries over 25 Years. N. Engl. J. Med..

[7] Lu Y., Hajifathalian K., Ezzati M., Woodward M., Rimm E.B., Danaei G. (2014). Metabolic mediators of the effects of body-mass index, overweight, and obesity on coronary heart disease and stroke: A pooled analysis of 97 prospective cohorts with 1·8 million participants. Lancet.

[8] Stanford F.C., Tauqeer Z., Kyle T.K. (2018). Media and Its Influence on Obesity. Curr. Obes. Rep..

[9] Brown A.W., Ioannidis J.P., Cope M.B., Bier D.M., Allison D.B. (2014). Unscientific beliefs about scientific topics in nutrition. Adv. Nutr..

[10] Puhl R.M., Phelan S.M., Nadglowski J., Kyle T.K. (2016). Overcoming Weight Bias in the Management of Patients with Diabetes and Obesity. Clin. Diabetes.

[11] Zeng Q., Li N., Pan X.F., Chen L., Pan A. (2021). Clinical management and treatment of obesity in China. Lancet Diabetes Endocrinol..

[12] Anton S.D., Lee S.A., Donahoo W.T., McLaren C., Manini T., Leeuwenburgh C., Pahor M. (2019). The Effects of Time Restricted Feeding on Overweight, Older Adults: A Pilot Study. Nutrients.

[13] Anastasiou C.A., Karfopoulou E., Yannakoulia M. (2015). Weight regaining: From statistics and behaviors to physiology and metabolism. Metabolism.

[14] Harvie M., Wright C., Pegington M., McMullan D., Mitchell E., Martin B., Cutler R.G., Evans G., Whiteside S., Maudsley S. (2013). The effect of intermittent energy and carbohydrate restriction v. daily energy restriction on weight loss and metabolic disease risk markers in overweight women. Br. J. Nutr..

[15] Warren J.M., Smith N., Ashwell M. (2017). A structured literature review on the role of mindfulness, mindful eating and intuitive eating in changing eating behaviours: Effectiveness and associated potential mechanisms. Nutr. Res. Rev..

[16] Robinson E., Aveyard P., Daley A., Jolly K., Lewis A., Lycett D., Higgs S. (2013). Eating attentively: A systematic review and meta-analysis of the effect of food intake memory and awareness on eating. Am. J. Clin. Nutr..

[17] Dalen J., Smith B.W., Shelley B.M., Sloan A.L., Leahigh L., Begay D. (2010). Pilot study: Mindful Eating and Living (MEAL): Weight, eating behavior, and psychological outcomes associated with a mindfulness-based intervention for people with obesity. Complement Ther. Med..

[18] Salvo V., Kristeller J., Montero Marin J., Sanudo A., Lourenço B.H., Schveitzer M.C., D’Almeida V., Morillo H., Gimeno S.G.A., Garcia-Campayo J. (2018). Mindfulness as a complementary intervention in the treatment of overweight and obesity in primary health care: Study protocol for a randomised controlled trial. Trials.

[19] Wewege M., van den Berg R., Ward R.E., Keech A. (2017). The effects of high-intensity interval training vs. moderate-intensity continuous training on body composition in overweight and obese adults: A systematic review and meta-analysis. Obes. Rev..

[20] Schaun G.Z., Pinto S.S., Praia A.B.C., Alberton C.L. (2018). Energy expenditure and EPOC between water-based high-intensity interval training and moderate-intensity continuous training sessions in healthy women. J. Sports Sci..

[21] Santos A., Braaten K., MacPherson M., Vasconcellos D., Vis-Dunbar M., Lonsdale C., Lubans D., Jung M.E. (2023). Rates of compliance and adherence to high-intensity interval training: A systematic review and Meta-analyses. Int. J. Behav. Nutr. Phys. Act..

[22] Guo Z., Cai J., Wu Z., Gong W. (2022). Effect of High-Intensity Interval Training Combined with Fasting in the Treatment of Overweight and Obese Adults: A Systematic Review and Meta-Analysis. Int. J. Environ. Res. Public Health.

[23] Jones R.A., Lawlor E.R., Birch J.M., Patel M.I., Werneck A.O., Hoare E., Griffin S.J., van Sluijs E.M.F., Sharp S.J., Ahern A.L. (2021). The impact of adult behavioural weight management interventions on mental health: A systematic review and meta-analysis. Obes. Rev..

[24] Rand K., Vallis M., Aston M., Price S., Piccinini-Vallis H., Rehman L., Kirk S.F.L. (2017). “It is not the diet; it is the mental part we need help with.” A multilevel analysis of psychological, emotional, and social well-being in obesity. Int. J. Qual. Stud. Health Well-Being.

[25] Pila E., Sabiston C.M., Brunet J., Castonguay A.L., O’Loughlin J. (2015). Do body-related shame and guilt mediate the association between weight status and self-esteem?. J. Health Psychol..

[26] Mustapic J., Marcinko D., Vargek P. (2015). Eating behaviours in adolescent girls: The role of body shame and body dissatisfaction. Eat. Weight Disord..

[27] Imanaka M., Ando M., Kitamura T., Kawamura T. (2013). Effectiveness of web-based self-disclosure peer-to-peer support for weight loss: Randomized controlled trial. J. Med. Internet Res..

[28] Lisón J.F., Palomar G., Mensorio M.S., Baños R.M., Cebolla-Martí A., Botella C., Benavent-Caballer V., Rodilla E. (2020). Impact of a Web-Based Exercise and Nutritional Education Intervention in Patients Who Are Obese with Hypertension: Randomized Wait-List Controlled Trial. J. Med. Internet Res..

[29] Múzquiz-Barberá P., Ruiz-Cortés M., Herrero R., Vara M.D., Escrivá-Martínez T., Baños R.M., Rodilla E., Lisón J.F. (2023). “Own doctor” presence in a web-based lifestyle intervention for adults with obesity and hypertension: A randomized controlled trial. Front. Public Health.

[30] Shi Y., Wakaba K., Kiyohara K., Hayashi F., Tsushita K., Nakata Y. (2022). Effectiveness and Components of Web-Based Interventions on Weight Changes in Adults Who Were Overweight and Obese: A Systematic Review with Meta-Analyses. Nutrients.

[31] Kohl J., Brame J., Centner C., Wurst R., Fuchs R., Sehlbrede M., Tinsel I., Maiwald P., Fichtner U.A., Armbruster C. (2023). Effects of a Web-Based Lifestyle Intervention on Weight Loss and Cardiometabolic Risk Factors in Adults with Overweight and Obesity: Randomized Controlled Clinical Trial. J. Med. Internet Res..

[32] Sakane N., Suganuma A., Domichi M., Sukino S., Abe K., Fujisaki A., Kanazawa A., Sugimoto M. (2023). The Effect of a mHealth App (KENPO-app) for Specific Health Guidance on Weight Changes in Adults with Obesity and Hypertension: Pilot Randomized Controlled Trial. JMIR Mhealth Uhealth.

[33] Conradt M., Dierk J.M., Schlumberger P., Rauh E., Hebebrand J., Rief W. (2007). Development of the Weight- and Body-Related Shame and Guilt scale (WEB-SG) in a nonclinical sample of obese individuals. J. Pers. Assess..

[34] Jiang C., Zhu Y., Luo Y., Tan C.S., Mastrotheodoros S., Costa P., Chen L., Guo L., Ma H., Meng R. (2023). Validation of the Chinese version of the Rosenberg Self-Esteem Scale: Evidence from a three-wave longitudinal study. BMC Psychol..

[35] de Lauzon B., Romon M., Deschamps V., Lafay L., Borys J.M., Karlsson J., Ducimetière P., Charles M.A. (2004). The Three-Factor Eating Questionnaire-R18 is able to distinguish among different eating patterns in a general population. J. Nutr..

[36] Khalafi M., Symonds M.E., Maleki A.H., Sakhaei M.H., Ehsanifar M., Rosenkranz S.K. (2024). Combined versus independent effects of exercise training and intermittent fasting on body composition and cardiometabolic health in adults: A systematic review and meta-analysis. Nutr. J..

[37] West D.S., Krukowski R.A., Finkelstein E.A., Stansbury M.L., Ogden D.E., Monroe C.M., Carpenter C.A., Naud S., Harvey J.R. (2020). Adding Financial Incentives to Online Group-Based Behavioral Weight Control: An RCT. Am. J. Prev. Med..

[38] Fichtner U.A., Armbruster C., Bischoff M., Maiwald P., Sehlbrede M., Tinsel I., Brame J., Kohl J., König D., Fuchs R. (2022). Evaluation of an Interactive Web-Based Health Program for Weight Loss-A Randomized Controlled Trial. Int. J. Environ. Res. Public Health.

[39] Brindal E., Freyne J., Saunders I., Berkovsky S., Smith G., Noakes M. (2012). Features predicting weight loss in overweight or obese participants in a web-based intervention: Randomized trial. J. Med. Internet Res..

[40] Muscogiuri G., Verde L., Vetrani C., Barrea L., Savastano S., Colao A. (2024). Obesity: A gender-view. J. Endocrinol. Investig..

[41] Sun S., Kong Z., Shi Q., Hu M., Zhang H., Zhang D., Nie J. (2019). Non-Energy-Restricted Low-Carbohydrate Diet Combined with Exercise Intervention Improved Cardiometabolic Health in Overweight Chinese Females. Nutrients.

[42] Catenacci V.A., Ostendorf D.M., Pan Z., Bing K., Wayland L.T., Seyoum E., Stauffer B.L., Phelan S., Creasy S.A., Caldwell A.E. (2019). The Impact of Timing of Exercise Initiation on Weight Loss: An 18-Month Randomized Clinical Trial. Obesity.

[43] Lupis S.B., Sabik N.J., Wolf J.M. (2016). Role of shame and body esteem in cortisol stress responses. J. Behav. Med..

[44] Turk F., Kellett S., Waller G. (2023). Testing a Low-Intensity Single-Session Self-Compassion Intervention for State Body Shame in Adult Women: A Dismantling Randomized Controlled Trial. Behav. Ther..

[45] Anderson L.M., Hall L.M.J., Crosby R.D., Crow S.J., Berg K.C., Durkin N.E., Engel S.G., Peterson C.B. (2022). “Feeling fat,” disgust, guilt, and shame: Preliminary evaluation of a mediation model of binge-eating in adults with higher-weight bodies. Body Image.

[46] Gao Z., Zhao J., Peng S., Yuan H. (2024). The Relationship and Effects of Self-Esteem and Body Shape on Eating Disorder Behavior: A Cross-Sectional Survey of Chinese University Students. Healthcare.

[47] Conlin L.A., Aguilar D.T., Rogers G.E., Campbell B.I. (2021). Flexible vs. rigid dieting in resistance-trained individuals seeking to optimize their physiques: A randomized controlled trial. J. Int. Soc. Sports Nutr..

