# Supplementary material for: The Effect of Online Fitness Combining Dietary Intervention on Body Composition, Body Shame and Self-Esteem in Young Adults: A Randomized Controlled Trial
Source: Nutrients. 2026 May 2;18(9):1460. doi: 10.3390/nu18091460 (PMC13164652; doi:10.3390/nu18091460)
Supplement: Supplementary file 1 [file nutrients-18-01460-s001.zip › nutrients-4247371-supplementary.pdf]

Supplementary Table S1. HIIT sessions.

Week	Duration of each segment	Work-to-rest ratio	Number of sets	Exercises
1-2	30s	2:3	4	1. Jumping jacks<br>2. Squats<br>3. Lunges<br>4. High knees
3-6	30s	1:1	5	1. Spiderman push-up<br>2. Squat jumps<br>3. Lunges<br>4. Fast high knees
7-8	30s	1:1	5	1. Burpees<br>2. Plank with shoulder taps<br>3. Squat jumps<br>4. Fast high knees

Supplementary Table S2. Core Principles of the 16/8 Intermittent Fasting and Mindful Eating Approach.

Number	Session content
Session 1	Introduction to core principles of mindful eating and 16/8 Intermittent Fasting
Session 2	Theme: Hunger cues-physiology v. emotional, hunger awareness, eating exercise and self-touch
Session 3	Theme: Taste satiety cues-type and level of cues, prioritize feeling comfortably satisfied than overly full
Session 4	Theme: Each meal should comprise 50% vegetables, 25% lean protein, and 25% complex carbohydrates
Session 5	Theme: How to combine mindful eating with 16/8 Intermittent Fasting
Session 6	Theme: Moderation and flexibility-adjustable eating window and occasional treats in moderation

Supplementary File S1: Daily Dietary Adherence and Satisfaction Questionnaire

1. Before your first meal today, did you feel hunger? Yes/ No
2. Did you complete all your meals within an 8-hour eating window today? Yes/ No
3. If you were unable to complete your meals within the 8-hour window, what was the primary reason?
   - Engaged in academic or work tasks, unable to complete meals on time.
   - Experienced strong cravings, leading to additional intake.
   - Forgot or overlooked the time restriction.
   - Inadequate meal planning led to hunger later, making it difficult to adhere.
   - Other (please specify): _______
   - I adhered to the protocol.
4. How satisfied are you with your adherence to the 16/8 intermittent fasting protocol today?

(Please rate your satisfaction on a scale of 1 to 10, where 1 is "very unsatisfied" and 10 is "very satisfied.")

Supplementary File S2: Weekly Exercise Adherence and Satisfaction Questionnaire

1. Did you complete the prescribed High-Intensity Interval Training (HIIT) sessions this week according to the program guidelines? Yes/ No
2. If you were unable to complete the prescribed sessions, what was the primary reason?
   - Lack of time due to academic/work obligations.
   - Physical fatigue or discomfort.

- Lack of motivation.
- Forgot or did not prioritize the sessions.
- Other (please specify): _______
- I completed all sessions as instructed.

3. How satisfied are you with your adherence to the exercise regimen this week?

   (Please rate your satisfaction on a scale of 1 to 10, where 1 is "very unsatisfied" and 10 is "very satisfied.")
